# Embryo donation: Survey of *in-vitro* fertilization (IVF) patients and randomized trial of complimentary counseling

**DOI:** 10.1371/journal.pone.0221149

**Published:** 2019-08-15

**Authors:** Alison E. Zimon, Donald S. Shepard, Jeffrey Prottas, Kristin L. Rooney, Jeanie Ungerleider, Yara A. Halasa-Rappel, Denny Sakkas, Selwyn P. Oskowitz

**Affiliations:** 1 Boston IVF, Waltham, Massachusetts, United States of America; 2 Heller School for Social Policy and Management, Brandeis University, Waltham, Massachusetts, United States of America; Universite Clermont Auvergne, FRANCE

## Abstract

**Design:**

This study surveyed patients with stored frozen embryos and developed and tested an intervention through a randomized trial to support subjects to consider embryo disposition options (EDOs), especially donation for family building.

**Methods:**

Based on a review of literature on EDOs, the authors developed and mailed a 2-page anonymous survey to 1,053 patients in Massachusetts (USA) to elicit their feelings about their stored embryos. Target patients had embryos cryopreserved for ≥1 year and had not indicated an EDO. Survey respondents were next randomized between usual care (control arm) or an offer of complimentary counseling and educational support regarding EDOs. These counseling sessions were conducted by a licensed mental health professional specializing in infertility treatment.

**Results:**

Despite telephone reminders, only 21.3% of patients responded, likely reflecting most patients’ reluctance to address EDOs. Respondents endorsed an average of 2 of the 5 EDOs, with the following percentages supporting each option: store for future attempts (82%), continue storage (79%), donate to research (29%), discard (14%), and donate for family building (13%). When asked their opinions towards embryo donation to another couple, 78% of patients agreed that donation is a way to help another couple, 48% would consider embryo donation to another family if they had a better understanding of the process, and 38% would be willing to consider donation if they were not going to use the embryos themselves, but 73% expressed discomfort with donation. In the randomized trial, 7.8% of intervention subjects (n = 8) obtained counseling sessions compared to 0.0% (none) of usual care subjects (p = 0.0069). Counseling participants valued not only discussing EDOs, but also assistance in expressing their feelings and differences with their partners.

**Conclusion:**

Improvement in counseling rates over the control arm suggests that free professional counseling is a small, but likely effective, step towards deciding on an EDO.

ClinicalTrials.gov Identifier: NCT01883934 (Frozen embryo donation study).

## Introduction

In 2003, a study performed by the Society for Assisted Reproductive Technology (SART) and the RAND Corporation surveyed more than 430 assisted reproductive technology (ART) practices in the United States and reported that there were approximately 400,000 embryos stored in in-vitro fertilization (IVF) centers throughout the USA [1]. This number has probably risen to well over half a million, and is likely to grow dramatically with improved success of IVF, embryo culture, and cryopreservation techniques [2].

When patients decide they will not use their embryos to achieve a pregnancy, they are faced with a stressful decision as to what to do with the surplus embryos. For many, this decision is emotional and difficult [3, 4].

Unlike many other developed countries, the United States does not have a time limit on embryo storage. However, the American Society for Reproductive Medicine (ASRM) has issued periodic guidelines, most recently in 2019 [5]. By contrast, the UK and Australia limit storage to 5–10 years [6, 7]. Beyond those time limits, clinics are then legally allowed to discard embryos. In many US-based clinics, a consent may include wording limiting the duration of embryo storage; however, most clinics do not act on that limit out of concern about a possible lawsuit. As such, patients are usually offered four options for embryo disposition when they store embryos: (1) continue storage for future use, (2) donate to scientific research, (3) donate to another individual or couple for family building, or (4) discard. While not listed as a formal option, the fifth option, defaulted to by many patients, is to avoid making a decision all together [4, 8]. For other patients, the financial burden motivates them to face the issue. In addition, couples may have differing views about these options.

Continued embryo storage presents problems for both patients and clinics. For patients, continued storage may lead to ongoing emotional and financial conflict. Clinics must invest material resources (e.g., liquid nitrogen), human resources, and infrastructure (e.g., equipment and alarm systems) to keep the frozen embryos safe and accurately documented, and to solicit disposition decisions. Failure to provide these resources adequately can be disastrous. One equipment malfunction destroyed 2,000 embryos and devastated hundreds of couples [9]. After the first year, clinics generally charge patients a fee for storing embryos to cover these expenses, typically $350 to $1,000 per year [10].

Donation of frozen embryos to a woman or couple for family building has several advantages [11]. The donor embryos have already been created, are often derived from a cohort of embryos with proven success, and provide an altruistic opportunity to help the infertile. Clinics invest time and support to facilitate embryo donation. Once prospective donors’ concerns towards embryo donation are known, clinics can support patients considering their embryo disposition options. Since 2002, the United States Congress has funded the Frozen Embryo Adoption Public Awareness Campaign [12] “to increase public awareness of embryo donation/adoption” and” fund projects that provide services to make this family building option more attainable for infertile couples” [12].[13] Accordingly, this study sought to assess patients’ attitudes and then to develop, implement and evaluate an intervention to support patients in considering EDOs, including those for family building. As the first step, we reviewed research and documents on EDOs in the United States and internationally at the time of the study’s initiation (mid-2013). The form completed by patients at the study site (Boston IVF) to document their EDOs (written prior to this study) is shown in S1 and S2 Files. [Patient’s declaration of preferred EDO (in use in mid-2013) and Patient’s declaration of preferred EDO (mid-2018)].

### US research on EDOs by mid-2013

Embryo donation for family building is an option for patients who wish to give their embryos a chance at life but do not wish to, or are not able to use them on their own. Unfortunately, embryo donation is underutilized and the demand for embryos is higher than the supply. One limitation is the considerable variation in rules and policies surrounding embryo donation from state to state, as well as worldwide. In addition, the fact that IVF centers generally do not have an assured supply of donated frozen embryos can make them unwilling to actively bring this option to the attention of patients.

A US study comparing patients using their own oocytes versus donor oocytes reported those using donor oocytes were more willing to donate to another couple and less likely to discard. Not surprisingly, this population may feel more desire to help future couples. However, there are often time limitations on the donor oocyte contract which restrict donation to another individual. While patients were favorable to the idea of donating their embryos to an infertile individual or couple, they often emphasized it was not something they personally could do. There were cultural, religious, spiritual, psychological, and ethical aspects to consider before making the decision to donate embryos to another person [14].

A study on US national trends and outcomes of embryo donation from 2000 through 2013 found that the annual number of donor embryo transfers increased exponentially from 332 to 1,374. However, donor embryo transfers as a share of all frozen embryo transfers remained low and essentially unchanged over this period (2.3% to 2.6%) [11].

According to the ASRM guidelines, an embryo may be considered abandoned if at least five years have passed since contact with the patient, numerous efforts have been made to contact the patient, and there are no written instructions from the patient concerning embryo disposition [15]. One study noted that of 3,888 couples with frozen embryos, more than half could not be contacted to make a disposition decision [8].

### International experiences on EDOs by mid-2013

In contrast to the US, several European countries and Australia have laws in place to limit the long-term storage of frozen embryos. These and other policies have favored research on EDOs. In the UK, for example, the normal maximum period that frozen embryos can be stored, according to the Human Fertilisation and Embryology Authority, is two terms of five years [7]. This period may be extended depending on the medical circumstances of the woman undergoing treatment, her partner and/or a donor.

A Canadian study based on structured interviews with 33 IVF couples who had embryos frozen in storage for three years found that these patients were not well informed of their EDOs [3].

A survey in Portugal found that younger patients (under 36 years) who viewed embryo research as very important and Catholic men were more willing to donate, while men with high levels of trait anxiety and those who view embryos as human beings were less likely to donate to research [4].

Overall, few patients seem to be interested in donation to another couple: a survey conducted by McMahon and Saunders of 133 Australian patients showed 74% were not interested in donation to another couple [4] which is comparable to our finding of 62%. Similar to the written comments on the present study, McMahon indicated reluctance to donate was related to a view of the embryo as a direct sibling to existing children, the feeling of ongoing responsibility for the well-being of the offspring, and the desire to know the characteristics of the recipients [4]. A survey of Swedish patients found the majority of respondents were interested in embryo donation to another couple; however, this form of donation is not legal in that country [16, 17]. A Belgian study of 412 patients described patients declining to make an embryo disposition decision as older, out of treatment longer, and had experienced more negative outcomes than patients making decisions [18].

Additional Belgian research documented a two-stage process of decision making: first, helping the patient to understand the medical procedure, and second, addressing patients’ emotional attachment to their embryos, with the embryos becoming a “symbol of one’s relationship”and their disposition sometimes conferring grief [19]. [18, 19]In a 15-year study on trends in EDOs, the same authors found that decisions changed over time, particularly when legally allowed to add a choice for donation to embryo research [20]. This choice then become the most popular, while discarding embryos and donation to other couples declined. The authors also reported that patients’ decision-making was a very private matter, seldom reaching out to clinic staff for guidance [21]. On-line anonymous support groups of patients in the same situation suggested that social media platforms were an easier way to connect people with like-minded attitudes and trends while preserving privacy [21].

Provoost provided two suggestions of how clinics can support patients in the embryo disposition process which contributed to the development of the intervention portion of our study: 1) offer support and counseling, and 2) remain in steady contact with the patients who have frozen embryos. Provoost also recommended that clinics ask patients to make an embryo disposition decision in advance [18].

Researchers in China [22] interviewed 363 couples who already had biological children but who still had frozen embryos in storage. These patients tended to wait until their children were older than 3 years before making a decision, and favored discarding embryos over donation to research due to lack of information on the clinical research being conducted.

### Embryo donation to research

While this study’s funder was particularly interested in embryo donation for family building, we also understood that our research and IVF patients needed to address all EDOs, including discarding embryos and donation to research. Patients decide to donate to research for a number of reasons: to support scientific research, to give their embryos a purpose, or because they simply cannot decide on any of the other options. The way patients think about the embryo may impact their decision to donate to research [23, 24]. If patients view the embryo as a “project,” they are more likely to donate to research [25]. Patients generally had uncertainties [26] about donation and ultimately chose not to donate due to lack of information or fears about embryo research [27].

### Need for empirical research and randomized trial

This literature review found that in both the United States and other countries, patients were uncomfortable considering and discussing EDOs. However, the Massachusetts’ policy environment could result in different patient attitudes compared to other states [28]. As of mid-2013, Massachusetts had some of the most comprehensive insurance coverage for ART in the US. In light of this public policy, patients in Massachusetts might be more receptive to embryo donation than those in other locales. The literature found no evidence of prior randomized trials of interventions to increase interest in embryo donation options. This gap suggested that a randomized trial would be a useful contribution to policy in this area, testing whether an intervention (complimentary psychosocial consultation with a licensed mental health professional at Boston IVF) would increase willingness to explore EDOs. A survey could help determine the types of patients who might be most receptive to exploring EDOs.

Therefore, this study next surveyed patients with stored embryos to assess their knowledge about EDOs, their willingness to donate to others for family building, and factors related to their willingness to donate. It finally tested the hypothesis through a randomized trial that the offer of a complimentary counseling session would increase the participation rate in sessions to discuss EDOs in general, and for donation to family building specifically.

## Materials and methods

### Study design

Following a publicly registered protocol (NCT01883934), this study was designed with a mixed quantitative and qualitative approach. It used a descriptive questionnaire-based survey of a large sample of subjects with frozen embryos stored for at least one year. Patients who responded to the survey were subsequently randomized to be offered complimentary counseling and educational support regarding EDOs or usual care. Separate from this study, the legal documents around embryo storage and distribution were signed as applicable by both partners with responsibility for the embryos.

### Study population

The study was based at Boston IVF, a high-volume infertility practice based in Waltham, MA and an affiliate of Beth Israel Deaconess Medical Center and Harvard Medical School. As Boston IVF is located in Massachusetts, a state with an insurance mandate for infertility coverage, 85% of Boston IVF’s patients have insurance coverage for infertility treatment. Most embryos in storage at Boston IVF date from 2005 onward. A total of 1,053 individuals with embryos in long-term storage (one year or longer) dating from January 2005 to 2013 were identified as eligible for the survey. The survey was completed in 2012–2013. For this study and most communications, Boston IVF linked each embryo to a single patient, almost always a woman.

### Procedure

The survey was mailed to all 1,053 eligible patients. The mailing included a personalized cover letter from the patient’s physician of record explaining study’s purpose and funding source, and emphasized that participation was voluntary and confidential. See S3 File [Letter to sampled patients from patient’s physician of record]. Having the letter come from the patient’s physician endeavored to strengthen communication. Finally, the study coordinator telephoned patients who had not yet responded 4 and 8 weeks after the initial mailing to remind them of the survey and to ask them to return the completed questionnaire.

This transmittal letter (S3 File) from the patient’s physician served as the information document for patients. It identified the study, explained its purpose, the burden to potential respondents (about 20 minutes’ time), the expected benefit to the clinic of the information requested, and the safeguards (voluntary participation, anonymous data, and used only for statistical analysis). Patients voluntarily mailing back their completed questionnaires constituted their informed written consent to participate in the survey phase of the study. These procedures followed those approved by the ethical review committee, as described below.

### The survey

The survey consisted of 19 questions about family knowledge, attitudes regarding embryo disposition, and factors affecting preferences. It also included eight demographic and background questions (S4 File [Questionnaire for surveying Boston IVF patients with embryos in storage].

The questions were designed to assess subjects’ attitudes, opinions and concerns that had been identified through the literature review. As the literature indicated that patients may have strong emotions and conflicting concerns, we structured most questions with a five-point Likert scale [29]. The numbers and corresponding response labels were (1) “strongly disagree,” (2) “disagree, (3) “neutral” or “don’t know,” (4) “agree,” and (5) “strongly agree.” Realizing that respondents could be considering multiple options, these alternatives were not exclusive.

The questions were grouped into three areas. The first set of questions asked which disposition options the subject would consider at the time of the survey; the options included ongoing storage for future use, ongoing storage for a future decision to discard, donation to research, or donation to another couple. The second set of questions addressed a number of personal scenarios and asked which scenario would make the subject more or less likely to donate embryos. Based on the prior literature, the survey asked separately about general attitudes towards embryo donation (questions B1-B7) as well as the respondent’s specific plans (questions A1-A5). The third set invited open ended comments to capture further concerns and emotions insufficiently addressed in the previous items. S5 File [Data set for survey respondents] and S6 File [Code book for data set].

### Proactive counseling intervention

Patients were randomized if they had been eligible for and responded to the survey, did not request to withdraw, and had not made a decision about embryo disposition at the time of randomization in mid-2013. In order to maximize precision of our estimate, all eligible patients were randomized. Using a parallel design, eligible survey respondents were randomly allocated 1:1 between control and intervention arms using a computer-generated random number under the guidance of the Brandeis investigators. Following the original design, the intervention group’s study numbers were linked to their home addresses and used to address envelopes. These were used to mail a letter, signed by the patient’s doctor of record (S7 File [Letter to intervention patients from senior study physicians]). The letter conveyed the offer of a complimentary counseling session and educational support regarding EDOs, including embryo donation. Recipients were invited to contact the site to schedule their completely voluntary, complimentary, confidential counseling session. There was no blocking or stratification and no other changes to patients’ care or financial obligations. This letter to intervention patients from the senior study physicians (S7 File) served as the information document for patients for the intervention phase of the study. Patients’ voluntary engagement in the counseling session constituted their verbal consent to participate in this phase of the study.

Based on the depth of emotion displayed in the open-ended survey responses as well as the need for privacy, Boston IVF designated an experienced, licensed mental health professional, rather than a peer, to conduct the counseling sessions. The sessions were confidential, flexible in schedule and free of charge to the patient. The usual care group remained eligible for all services in the IVF practice, but the patient needed to initiate any service request and make or arrange payment. The outcome measure consisted of documentation of a request for a counseling session with a licensed mental health professional to discuss EDOs in a services log within 12 months of randomization. The study’s research coordinator reviewed these logs; other researchers were blinded. No additional outcomes were measured.

### Analysis

First, numerical data and Likert scale questions were entered into a spreadsheet. Likert scores of 1 and 2 were classified as “disagree,” 3 as “don’t know” and 4 and 5 as “agree” and percentages were calculated. As a basic examination of the responses to open-ended questions, we used Wordcloud.com to tabulate frequencies of word roots (e.g. combining donate and donation) to identify salient terms.

Second, we counted the qualitative responses in relation to the answers to the respondent’s level of support for survey question A3 “Donate to another couple trying to have a baby.” Third, we examined how the male perspective was reflected in survey responses. While almost all eligible patients were women, their responses could potentially incorporate their partners’ views. While the survey did not ask about this explicitly, we inferred this perspective insofar as possible by analyzing personal pronouns in the open-ended responses, (i.e., I, we, my, mine, our, and us). Among responses containing any personal pronouns, we counted the share using a plural form.

Fourth, three authors (DSS, DS and JU) applied Grounded Theory, an inductive process, to these written comments [30]. These three authors of the current study identified key phrases, noted their recurrence across respondents, and identified key themes and constructs. They then selected representative extracts as explanations.

Fifth, as an exploratory analysis, we tabulated donation for family building against use of donor gametes. We analyzed the results of this exploratory analysis and the trial component for participation in counseling sessions for statistical significance with a two-sided Fisher’s exact test due to its higher accuracy with anticipated small cell sizes [31] and 95% confidence intervals were computed with Wilson’s procedure with the continuity correction [32, 33]. No other interim, subgroup, exploratory or adjusted analyses were planned or conducted on Likert scale items.

### Ethical review

The research was a collaboration between researchers at Boston IVF and The Heller School for Social Policy and Management at Brandeis University. The study was approved by the Committee for the Protection of Human Services at Brandeis University and implemented according to the committee’s guidelines. See S8 File [Final version of the approved protocol]. As noted previously, the cover letter from the patient’s physician of record (S3 File) explained that participation in the study was voluntary and confidential. Likewise, the letter from the senior study physicians (S7 File) explained that participation in the counseling session was voluntary and confidential.

## Results

### Patient demographics and patient flow

**Fig 1:**
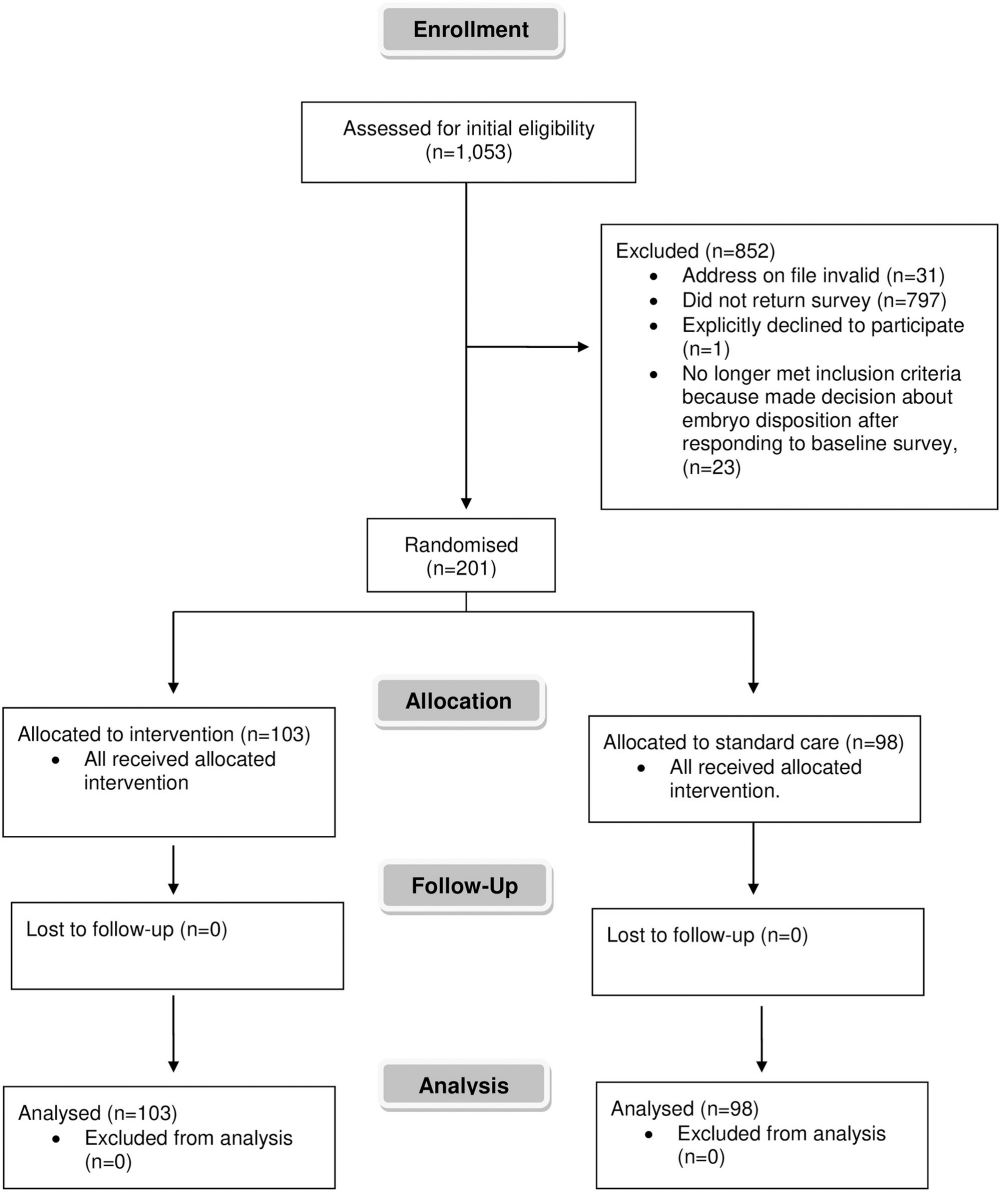
CONSORT patient flow diagram.

The survey was sent to 1,053 subjects with embryos in storage at Boston IVF for over one year. A total of 224 subjects submitted a completed survey for a response rate of 21.3%. Almost all respondents (215 out of 224) were female (Table 1). They were predominantly college educated, had children, Caucasian race, and gave a religious affiliation. Their demographic characteristics are a reasonable reflection of Boston IVF’s patient pool. Fig 1 shows the CONSORT diagram with patient flow for the study as a whole (S9 File).

**Table 1:** Characteristics of the 224 survey respondents showing numbers of responses (n) and percentages (%).

Characteristic	n	(%)
**Female**	215	(96%)
**Have children**	213	(95%)
**College education or more**	206	(92%)
**Religion**		
** Protestant**	52	(23%)
** Catholic**	94	(42%)
** Jewish**	18	(8%)
** Other**	63	(28%)
**Caucasian race**	197	(88%)
**Used donor gametes**	60	(27%)

### Embryo disposition: Likert scale responses

With respect to specific embryo disposition plans of the responding subjects in a multi-response format, 82% agreed with storing for future attempts and 79% agreed with continuing to store and deferring a disposition decision until a later time (Table 2). Fourteen percent of all respondents agreed with discarding, 29% agreed with donating to research, and only 13%, the lowest of all items, agreed with donating for family building. Even this small share represents 30 survey respondents who would consider donating to another patient for family building. The sum of these favorable responses (203%) indicates that the average respondent favored two choices, reflecting the conflicts around these dispositions.

**Table 2:** Disposition choices of the 224 survey respondents showing numbers of responses and row percentages (%).

What disposition would you choose at this time?	Disagree	Agree	Don’t know or no response
**Store for future attempts**	33 (15%)	184 (82%)	7 (3%)
**Keep for future decision**	27 (12%)	177 (79%)	20 (9%)
**Donate for research**	83 (37%)	65 (29%)	76 (34%)
**Discard**	148 (66%)	31 (14%)	45 (20%)
**Donate for family building**	140 (62%)	30 (13%)	54 (24%)

When asked about their thoughts towards embryo donation for family building, 78% of respondents agreed that donation is a way to help another couple (Table 3). However, 73% expressed discomfort with someone else raising their genetic child and 81% found embryo donation complex and difficult. Only 6% felt that donation-related expense or medical testing were significant concerns.

**Table 3:** Baseline attitudes of the 224 survey respondents towards embryo donation for family building showing numbers of responses and row percentages (%).

Thinking about donation for family building	Disagree	Agree	Don’t know or no response
**Donation is a way to give a chance of life**	54 (24%)	125 (56%)	45 (20%)
**Donation is a way to help another couple**	22 (10%)	175 (78%)	27 (12%)
**Donation is similar to adoption**	54 (24%)	123 (55%)	47 (21%)
**I am uncomfortable with someone else raising my genetic child**	31 (14%)	164 (73%)	29 (13%)
**Donation to a family is complex and difficult**	25 (11%)	181 (81%)	18 (8%)
**Donation expenses are a concern**	157 (70%)	13 (6%)	54 (24%)
**Testing or more medical procedures concerning donation are a concern**	150 (67%)	13 (6%)	61 (27%)

When asked what factors or scenarios may make these individuals more willing to donate to a family, 38% responded positively if they knew definitively that they did not want more children, and 48% responded positively if they understood the legal processes and implications of donation (Table 4). Respondents indicated their willingness to donate to a family if they could speak with a counselor (27%), would know the outcome of the donation (25%), would know the recipients (20%), or could ensure that the recipients were far from them geographically (12%).

**Table 4:** Factors related to a propensity to donate embryos of the 224 survey respondents showing numbers of responses and row percentages (%).

Might you be more willing to donate to a family if:	Disagree	Agree	Don’t know or no response
**I was sure I did not want more children**	105 (47%)	85 (38%)	34 (15%)
**I understood the legal implications**	107 (48%)	72 (32%)	45 (20%)
**I could speak with a counselor**	115 (51%)	60 (27%)	49 (22%)
**I would be informed of the outcome of the donation**	116 (52%)	56 (25%)	52 (23%)
**I would be informed of the basic characteristics of the recipients**	132 (59%)	47 (21%)	45 (20%)
**I would be in contact with the recipients**	123 (55%)	45 (20%)	56 (25%)
**The recipients lived in a different area or state**	116 (52%)	26 (12%)	82 (36%)

Most respondents were unwilling to dispose of stored embryos in any way, with over 80% favoring continuing storage. Among patients not planning to use their embryos, research use had twice the level of support as any alternative disposition. Interestingly, support for donation for family building was comparable to support for discarding. Although the 13% reporting interest in embryo donation presents a minority of patients, this share still represents a notable number of patients in the IVF practice. If this 13% share were representative of the clinic’s overall practice, it would translate to 136 of the 1,053 patients in Boston IVF’s practice with frozen embryos and perhaps 78,000 of 600,000 patients with stored embryos nationally.

### Embryo disposition: Open-ended responses

In the portion of the questionnaire inviting open-ended comments, a substantial share of respondents (83 of 224 or 37%) provided written responses. The length of these open-ended responses (often a paragraph) and their personal content evidenced the respondents’ depth of attachment to the embryos. Each comment averaged 51 words or about 5 sentences. The word count found that the top ten word roots (and associated frequencies were: embryo (122), donate (113), child (49), think (32), decision (30), couple (28), another (26), family (25), fertile (24), and frozen (23). Among these 83 respondents, 30% were favorable towards donation, 13% undecided or non-responsive, and 57% were unfavorable.

From the Grounded Theory, the first major theme was the tension between the support for embryo donation in the abstract, contrasted with the reluctance to allow another couple to have potential biological children and full siblings to the donor’s existing children. These are reflected in the following comments. Respondent 1: “I believe embryo donation is an amazing gift to give a couple or individual the chance to have a child that otherwise would not be able to. I think it is an incredibly selfless act to be able to donate. I, however, would not feel comfortable with a biological child out there in the world being raised by anyone other than me or my husband.” Respondent 2: “I am honestly glad that we used all our frozen embryos in this last attempt… I understand that donating embryos helps families, but I just wouldn’t be comfortable with it.” Respondent 3: “I am struggling to just thaw and discard as I would want the embryo to be put towards something useful.”

The next major theme was a complete rejection of the embryo donation, as reflected the following comments: Respondent 4: “I guess that some people are willing to donate their embryos for medical research. I couldn’t do it. I couldn’t do it because that’s not why I created the embryos.” Respondent 5: “My reservation about donating my embryo to another family is that it would be a sibling to my daughter, which I think would be unfair to her.”

A third theme was a reiteration of how precious the embryos were, leading to the respondent’s desire to continue storage. This was reflected by the following comments: Respondent 6: “I only have one frozen embryo and I am in the process of trying to use that one. I do feel like it is part of our family and should have a chance.” Respondent 7: “Personally, I struggle with the notion of donating my embryo mainly because I view it as my child and I’d want to raise it.”

The fourth theme was concerned with the financial burden of ongoing storage fees, as reflected by Respondent 8’s comments: “Keeping embryos in storage carries a huge emotional burden, mostly from indecision about what to do, and enormous financial responsibility.”

The final theme was support by a few respondents for donating their embryos, as reflected in the following comments: Respondent 9: “By far everything we went through on our journey to have children pales in comparison to the emotional upheaval in coming to the decision to donate our embryos to another couple. We are happy with our decision …” Respondent 10: “Donation takes a special person. We would not have our children if donors were not the selfless people they are. Respondent 11: “I feel that IVF/ART has given us a wonderful child/wonderful life that I would be willing to assist others achieve the same.” Respondent 12: “If I donate embryos, I’d want my children to have an opportunity for a relationship with any children that result from it.”

The analysis of pronouns found that 39% of the responses containing a personal pronoun (i.e. 31 of 80) contained a plural form (i.e., we, our or us). This tabulation indicates that many respondents were taking their partners’ feelings and preferences into account.

### Results of exploratory analysis related to donor gametes

The exploratory analysis found that 27% of patients with complete responses had used donor gametes. Of these patients, 19% agreed with donating to another family compared to only 11% of patients not using donor gametes. This pattern indicated that patients using donor gametes were somewhat more inclined towards donating to another family, but the difference was not statistically significant (p = 0.18).

### Intervention results

Of the 224 survey respondents, 23 had made a decision about embryo disposition by the time of randomization. The remaining 201 patients were randomized (103 to intervention and 98 to control). There were no losses or exclusions following randomization and all patients were analyzed based on originally assigned groups. Eight (7.8%) of the intervention patients accepted the offer of the complimentary counseling session (95% confidence interval 3.7% to 15.2%). None (0.0%) of the 98 control patients received a counseling intervention (95% confidence interval 0.0% to 4.7%). The difference between arms was highly significant (p = 0.0069). Because of the zero rate in the control group, the odds ratio was infinite.

The counselor reported that patients who participated in the counseling were extremely appreciative that the clinic offered the consultation. By being offered this session, patients felt that the medical team understood what a difficult decision this was for them. In the counseling session, they expressed relief to learn they were not alone in feeling so emotional about these options. Validating their feelings normalized their experience.

In some instances, female patients reported difficulty talking about the disposition of their frozen embryos because they had differing views from their partner. In some cases, the women expressed a wish to have another child, while their partner did not want more children. For some women, discussing the disposition of their embryos meant facing the reality that they had completed building their family and were coming to the end of their reproductive years. This led to a discussion of what it means for them to put closure on this stage in their life after years of struggling with infertility treatments. As no adverse effects of the trial of intervention were identified, the trial continued to its planned conclusion.

## Discussion

The survey confirmed that patients with embryos in storage remain emotionally attached to their embryos and conflicted about their disposition. Only 13% of the survey respondents reported wanting to donate their embryos to another patient for family building. While the 7.8% acceptance rate for complimentary counseling in the intervention arm was lower than previously expected, all recipients reported to the counselor that they found the service very gratifying. The counselor (JU) observed that patients found the discussions extremely valuable. When the two partners had different views, having an “outsider” facilitating the discussion made it possible for each of them to express their opinion.

We were unable to determine definitively the clients’ subsequent decisions as we did not request nor obtain permission to follow up on their choices. The counselor advised all counseling patients to take time to consider the decision carefully to make sure that the patient was comfortable with it. The counselor thinks there is a reasonable probability that at least one of the eight patients would decide to donate her embryos to another unrelated couple through an independent embryo donation service. Another patient indicated she planned to donate her embryos to research.

### Possible solutions

Tens of thousands of people seek fertility assistance every year from ART clinics. For those who cannot use their own oocytes, the options offered are limited. The most commonly used is to employ donor eggs. This is an effective option but costly and sometimes of limited availability. Given a large reserve demand and the presence of a large potential, but mostly untapped supply, potential options merit careful analysis.

A survey of 131 Canadian patients found that education on embryo disposition received after treatment was not adequate [34]. Bruno et al. suggested that providers need to counsel patients early in the IVF process, before a surplus of embryos has been created. Deciding to discontinue cryopreservation of their embryos proved significantly more difficult than choosing what to do with the embryos when they are created [25]. There is clearly a need for education of disposition options in the form of written information and counseling services both before and after treatment.

A California study [35] interviewed 106 families to understand their perception on how the IVF clinic handles EDOs. The patients wanted more written information reviewing their options, as well as someone at the clinic who could help coordinate logistics and offer support. Patients viewed embryo donation to research as an easier option compared to donation to another couple.

An Australian study interviewed 15 patients who completed embryo donation as donors or recipients. All the donors cited that they did not find the decision to donate difficult and neither donors nor recipients viewed the process as adoption [36].

### Ongoing counseling service at Boston IVF

Based on the literature, survey findings, and the favorable experience of patients receiving counseling sessions, Boston IVF decided to convert the study intervention into an ongoing counseling service. Starting in January 2014, Boston IVF modified its cover letter accompanying its invoice for the patient’s annual storage fee, initiating the offer of a complimentary counseling session as part of annual embryo storage for all patients. Under what the clinic calls its Frozen Embryo Donation Service (FREDS), Boston IVF sends patients with embryos in storage a letter three months ahead of the invoice for annual embryo storage inviting them to schedule this complimentary session (S10 File).

As of December 31, 2018, a period of four years since the start of the ongoing service, about 14,000 invitations were issued and 20 patients received such complimentary sessions, coming alone or as a couple. Following these sessions, the 20 patients continued to store their frozen embryos at Boston IVF (45%), discarded them (30%), donated them to research (10%), used them in their own treatment (10%), or transferred them to a long-term storage facility (5%). The acceptance rate for these sessions of 0.14% (95% confidence interval 0.08% to 0.21%) was considerably lower than that in the trial. Whereas the trial was limited to survey respondents, whose response demonstrated some engagement with the clinic around EDOs, FREDS was offered to all patients. Also, there was substantial overlap between patients in the trial and those eligible for FREDS, so the patients with the greatest interest may have already received counseling during the trial or a previous year.

A positive feature about the modest uptake to the counseling offer is that FREDS is an affordable service. We estimate the cost of providing a counseling session and associated overhead cost is about $200 per session. As the invitation was part of the same mailing invoicing the annual storage fee, there were no additional administrative costs. Boston IVF continues to employ staff responsible for responding to numerous telephone inquiries from patients about embryo storage and disposition. However, with the aforementioned acceptance rate of 0.14%, the added cost per patient with embryos in storage per year offered this counseling is only US$0.28 (i.e., 0.14% x $200).

The ASRM guidelines [15] suggest asking patients to document their decision regarding their frozen embryos before the embryos are even created. Patients may not be in the same emotional state before the embryos are created as they are once they actually have embryos. One study found only 29% of patients who made a choice for their embryos before treatment kept the same choice for their embryos after treatment [37].

### Study limitations

The main limitation of this study is that it was conducted in a single clinic (Boston IVF) and a single state (Massachusetts). Cultural differences between countries, states, and clinics could drastically alter perceptions to embryo disposition. Even within countries, such as the USA, widespread regional differences are apparent. As the state of Massachusetts, where this study was conducted, has mandated health insurance coverage for infertility treatment, the financial burden on this patient population may be less than in other US states and may influence their decisions on embryo disposition. In mandated insurance states, the level of IVF cycles performed per capita is four or more times higher than those without insurance coverage, hence any bias in access should be circumvented [38].

While the response rate to the survey was low (21%), we think this rate itself demonstrates patients’ ambivalence towards addressing EDOs. Additionally, the respondents’ characteristics in Table 1 indicate that they are similar to Boston IVF’s overall patient population based on the experience of the Boston IVF authors. As 96% of our respondents were female, our survey did not capture the male perspective directly. However, the language of a substantial share (39%) of the relevant open-ended responses referenced a partner. Thus, we feel that our survey responses reasonably reflected the male perspective.

The need to start our survey as promptly as possible after this project received funding allowed only a qualitative literature review when developing our questionnaire. Although we could not validate our Likert response items, our listing of salient root words and excerpts indicates that these respondents actively addressed the tensions around embryo donation.

It was not possible to ascertain whether patients discussed EDOs with medical professionals outside the counseling service, although the researchers were not aware of any such discussions. Only eight patients chose the free counseling option, hampering statistical analysis of this group.

## Conclusion

Patients are challenged to make a decision regarding EDOs. Despite the limitations, the study’s authors’ experience suggests a powerful benefit from professional social worker counseling over the difficulties inherent in choosing an EDO. A service such as FREDS is an important contributor to quality care at relatively minor cost and a potential contributor towards embryo donation.

## Supporting information

S1 File Consent for freezing embryos (mid-2013). This was the version of the form at Boston IVF in mid-2013, to be signed by both the male and female, to discard their frozen embryos.
(DOCX)

S2 File Consent for freezing embryos (mid-2018). This was the version of the form at Boston IVF in mid-2018, to be signed by both the patient and partner, to discard their frozen embryos.
(DOCX)

S3 File Letter to sampled patients from patient’s physician of record. This letter introduced the accompanying mailed survey in S4.
(DOC)

S4 File Questionnaire for surveying Boston IVF patients with embryos in storage. This questionnaire was mailed to sampled patients behind the cover letter in S3.
(DOCX)

S5 File Data set for survey respondents. This data set contains the responses to the questionnaire in S4.
(XLSX)

S6 File Code book for the data set. This document describes the codes and conventions in the data set S5.
(XLSX)

S7 File Letter to intervention patients from senior study physicians. This letter, sent to intervention patients, informed them about the availability of a complimentary counseling session.
(PDF)

S8 File Final version of protocol approved by the Brandeis University Committee for the Protection of Human subjects in research. (PDF)

S9 File CONSORT checklist for randomized trial. (DOCX)

S10 File Ongoing program letter. This letter to Boston IVF patients with frozen embryos in storage, instituted after the trial, informs them about the availability of a complimentary counseling session.
(DOCX)
