# Supplementary material for: Embryo donation: Survey of in-vitro fertilization (IVF) patients and randomized trial of complimentary counseling
Source: PLoS One. 2019 Aug 15;14(8):e0221149. doi: 10.1371/journal.pone.0221149 (PMC6695140; doi:10.1371/journal.pone.0221149)
Supplement: S2 File — This was the version of the form at Boston IVF in mid-2018, to be signed by both the patient and partner, to discard their frozen embryos. (DOCX) [file pone.0221149.s002.docx]

#
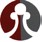
 BOSTON IVF

# CONSENT TO DISCARD Patient_____________________

**FROZEN EMBRYOS** Date of Birth ______________

We request that some or all of our cryopreserved (frozen) embryos no longer be stored at Boston IVF. We request that the embryos be thawed in the laboratory in a manner that will render them non-viable.

**Our instructions are as follows:**

1. **Choose one of the two options below regarding the number of embryos to be discarded:**

- 1. We desire that **ALL** of our embryos stored at Boston IVF be discarded.

___________ ___________

Patient’s Partner’s

Initials Initials

- 1. We desire that **ONLY** embryos frozen on **the following dates** be discarded:

**List dates of freeze (month/day/year) ________________________________**

___________ ___________

Patient’s Partner’s

Initials Initials

1. **Choose one of the three options below regarding the handling of the embryos after they are thawed**

- 1. We desire that Boston IVF discards the embryos according to their protocol.

___________ ___________

Patient’s Partner’s

Initials Initials

- 1. We donate our embryos for laboratory training and/or for research purposes aimed at improving IVF treatment outcome. If discarded embryos are studied as part of a research project it would only be done in compliance with Institutional Review Board (IRB) policy. All materials used for research purposes would be de-identified. No materials would be used to establish a pregnancy.

___________ ___________

Patient’s Partner’s Initials Initials

- 1. We wish to take the embryos with us for disposal as we see fit.

___________ ___________

Patient’s Partner’s

Initials Initials

Page 1 of 2 F-MD-1018 Rev 10

Copyright © 2016 by Boston IVF. All rights reserved.

##
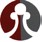
BOSTON IVF

We release the physicians, nurses, technicians, and other Boston IVF staff from any responsibilities regarding these embryos after they are thawed. We have had an opportunity to discuss this decision with the Boston IVF staff and understand the implications of this decision. We have been given the opportunity to ask questions which have been answered to our satisfaction in language that we understand by the staff of Boston IVF and we have considered alternative options.

**It is required that you have this document witnessed at Boston IVF, if unable because of distance the default is to have this document officially notarized.**

_____________________________ _____________________________

Signature of Patient Signature of Partner

_____________________________ _____________________________

Print Name Print Name

_____________________________ _____________________________

Date of Birth Date of Birth

_____________________________ _____________________________

Address Address

_____________________________ _____________________________

City/State/Zip City/State/Zip

_____________________________ _____________________________

Phone Phone

_____________________________ _____________________________

Email Email

_____________________________ _____________________________

Signature of BIVF Witness Signature of BIVF Witness

____­­______________________­­­___ _____________________________

Printed Name of BIVF Witness Printed Name of BIVF Witness

_____________________________ _____________________________

ID Verification (Type/#/Exp Date) ID Verification (Type/#/Exp Date)

_____________________________ _____________________________

Date of Consent Date of Consent

_______________________(State)

On this ____day of ____________, 201__, before me, the undersigned notary public, personally appeared _________________________, proved to me through satisfactory evidence of identification, which were _________________________, to be the personal whose name is signed on the proceeding or attached document in my presence.

_______________________________

Notary Public

_______________________(State)

On this ____day of ____________, 201__, before me, the undersigned notary public, personally appeared _________________________, proved to me through satisfactory evidence of identification, which were _________________________, to be the personal whose name is signed on the proceeding or attached document in my presence.

_______________________________

Notary Public

Page 2 of 2 F-MD-1018 Rev 10

Copyright © 2016 by Boston IVF. All rights reserved.
