# Supplementary material for: Embryo donation: Survey of in-vitro fertilization (IVF) patients and randomized trial of complimentary counseling
Source: PLoS One. 2019 Aug 15;14(8):e0221149. doi: 10.1371/journal.pone.0221149 (PMC6695140; doi:10.1371/journal.pone.0221149)
Supplement: S3 File — This letter introduced the accompanying mailed survey in S4. (DOC) [file pone.0221149.s003.doc]

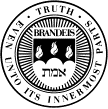


Dear Boston IVF Patient,

As part of a national study, Boston IVF and Brandeis University are conducting a survey of Boston IVF patients who have embryos in storage. The immediate goal is to better understand the attitudes and preferences of patients who have stored embryos. We will use this information to try to improve the practices of the IVF community both here in Boston and nationwide.

***Responding to the enclosed survey is voluntary and it is confidential.***

This survey will not take more than 20 minutes to complete. Your responses will be kept completely confidential. The answers will be anonymous; no individual answer will be connected to any person. Brandeis University will assist in the statistical analysis phase of the study. Brandeis University will not have access to patient identifications and the analysis will be purely statistical. Though supported in part with funds from the U.S. Department of Health and Human Services, this is **not** a government survey.

The findings will be used to help craft Boston IVF policy (and national policy) to better understand the views of the people who have embryos frozen in storage. These issues are complex and sometimes involve difficult decisions. This work cannot be done without your assistance.

While answering is completely voluntary, we hope you will spend the time needed. Your input will help Boston IVF and other clinics improve policies around frozen embryos.

Sincerely,

[Physician of Record]
