# Supplementary material for: Embryo donation: Survey of in-vitro fertilization (IVF) patients and randomized trial of complimentary counseling
Source: PLoS One. 2019 Aug 15;14(8):e0221149. doi: 10.1371/journal.pone.0221149 (PMC6695140; doi:10.1371/journal.pone.0221149)
Supplement: S7 File — This letter, sent to intervention patients, informed them about the availability of a complimentary counseling session. (PDF) [file pone.0221149.s007.pdf]

[Boston IVF letterhead]

Dear Patient,

We are contacting you as part of an initiative to support patients who have frozen embryos stored with Boston IVF.

You may be planning to use these embryos in the future, in which case you have likely decided to continue ongoing storage. If you are not planning to use your embryos in the future or have not yet made a decision, you may know that your options include ongoing storage, discard, donation to other individuals or families, or research donation. We recognize that these decisions are complex and information regarding your options may be difficult to obtain.

This service was initiated with the support of a grant from the U.S. Department of Health and Human Services (HHS). **We invite you to meet with the Director of Boston IVF's Counseling Services to explore and understand your options with respect to your frozen embryos. This consultation is free of charge.**

The consultation is conducted by Jeanie Ungerleider, LICSW, BCD, who is Boston IVF's Director of Counseling Services and has over twenty-five years of experience working with fertility patients at Boston IVF. The objective is to provide you with information and resources regarding embryo disposition as well as the support to deal with the difficult decisions regarding your embryos. Other Boston IVF patients have found these consultations extremely rewarding. Though this service has support from the Department of Health and Human Services, **it is not a government project** and the government will not receive any personal information about participants. There is no obligation to participate and your participation has no bearing on the status of your embryos or associated notifications or fees. This initiative will cover embryo storage fees going forward should you choose to donate your embryos to other individuals or families.

If you are interested in learning more about this initiative, please contact our **Program Coordinator at Boston IVF, Kristin Rooney, at 781-434-6470 between 7am and 3pm weekdays**. As always, your Boston IVF physician and team are available for support and information if you wish to further discuss your frozen embryo options.

Sincerely,

Selwyn P. Oskowitz, MD
Alison E. Zimon MD
Boston IVF Physicians
