# Supplementary material for: Embryo donation: Survey of in-vitro fertilization (IVF) patients and randomized trial of complimentary counseling
Source: PLoS One. 2019 Aug 15;14(8):e0221149. doi: 10.1371/journal.pone.0221149 (PMC6695140; doi:10.1371/journal.pone.0221149)
Supplement: S8 File — (PDF) [file pone.0221149.s008.pdf]

STUDY PROTOCOL

Part 1: Original protocol (2012)

a) Title and Principal Investigators:
 Provision of Frozen Embryo Donation Services in a Large IVF Clinic
 Alison Zimon for Boston IVF
 Don Shepard and Jeffrey Prottas for Brandeis University

Purpose:

b) Purpose
 Ten of thousands of infertile women seek help at clinics using assisted reproductive technologies (ART clinics). Some of these patients cannot become pregnant using their own oocyctes. Egg donation, the most common alternative, is expensive, uncomfortable and not always available. At the same time, patients of ART clinics have an estimated 500,000 viable embryos in frozen storage, but very few patients currently offer to donate these for the use of other patients. Boston IVF center will implement new services meant to increase the supply of donated embryos and to increase the awareness and interest in them among potential recipients. We propose a systematic evaluation effort to track and measure these activities.

Boston IVF will institute a protocol of enhanced services aimed at patients with embryos in storage to increase the number of such patients willing to consider donation. Using a randomization process, patients will be divided into two groups of 400 patients each: a control group who will receive ordinary services, and an intensive intervention group receiving more proactive outreach. All patients who express an interest will be offered enhanced counseling and educational services.

Boson IVF will also offer additional services to patients in its third party reproduction unit, consisting of an array of educational and counseling services and operational assistance to emphasize the option of frozen donated embryo use. The evaluation of these new services are closely integrated into the Boston IVF operational plans. Data will be gathered on donor attitudes and responses to the intervention. Recipients will be interviewed to measure increased knowledge and interest in donated embryos. Changes in patient behavior, including donation rates will be tracked. Changes in staff knowledge and attitudes will be studied.

c) Sponsor:

 Office of Population Affairs, Department of Health and Human Services

d) Principle Investigator(s)

 Alison Zimon, MD of the Boston IVF, is the project Principal Investigator and brings a rich experience in clinical knowledge, research and management. She is on faculty at Harvard Medical School as a clinical instructor and teaches medical students, residents and fellows. She has been the lead and co-author on peer reviewed papers within the fields of immunology, clinical gynecology, and ovarian basic science. She has received recognition and support for her research through a NIH Loan Repayment Program award and a Harvard Medical School Scholars award. At Boston IVF, she has spear-headed effort to introduce a clinical program for embryo donation. She has a strong interest in improving options and services for couples facing reproductive challenges. As principal investigator of this FREDS project, she plans surpass the challenges of embryo donation and allow this valuable option to become readily available for infertility patients.

At Brandeis the Co. PIs are Drs. Shepard and Prottas. Both have almost three decades of experience in health policy research and both have directed multi-site, complex research projects. Dr. Prottas is an expert on organizational behavior and implementation issues, as well as on the use of case studies and qualitative methodologies. Dr. Shepard is an expert on cost/benefit analysis and quantitative analytical techniques. Both have conducted and analyzed surveys. Last year Dr. Shepard and Dr. Prottas, working with the QED Group, conducted a study for the Office of Population Affairs entitled Study of Frozen Embryo Donation/Adoption Services. This project had several components and Brandeis' primary tasks dealt with the potential roles of ART clinics in increasing the use of donated frozen embryos. The work involved organizational analysis of 6 ART clinics across the nation and the modeling of the costs and benefits of new clinic based services. This work examined the parameters and issues arising from the introduction of new services at these clinics. Boston IVF was one of the IVF sites in the study. The Study of Frozen Embryo Donation and Adoption Services is substantively the most relevant prior work done by the evaluation team. However both of the senior Brandeis staff have other methodologically relevant experience. Dr. Shepard directed the evaluation of the CMS's "Demonstration Project on Life – Style Modification" and the evaluation of the "Illinois and Wisconsin State Pharmacy Assistance Waivers." Both were evaluations of operational experiments involving new interventions and both employed both survey and cost/benefit methodologies. Dr. Prottas directed the implementation components of the CMS's "Expansion of Medicare Coverage for Chiropractic Services." This evaluation included both interviews with providers and surveys of beneficiaries. A follow-up consisted of an extensive survey of providers focusing on the changes in clinical practices that followed from the expanded coverage under the Demonstration. Dr. Prottas now working a project addressing community health centers and how they manage efforts to coordinate care across organizational boundaries. This project too involves survey and case methods/

e. Student Researcher Qualifications: N/A

f. Other research personnel:

As this is a Demonstration grant directly with Boston IVF most personnel do not have an explicitly research role. The bulk of the activities are aimed a bringing new services to Boston IVF patients. At Brandeis, the following staff are joining the project with the responsibilities indicated. Aung Lwin (Research Associate) is performing survey analysis, and Wu Zeng (Scientist) and Yara Halasa (Research Associate) are performing cost analyses.

g. Previous work

Last year Dr. Shepard and Dr. Prottas, working with the QED Group, conducted a study for the Office of Population Affairs entitled Study of Frozen Embryo Donation/Adoption Services. This project had several components and Brandeis' primary tasks dealt with the potential roles of ART clinics in increasing the use of donated frozen embryos. The work involved organizational analysis of 6 ART clinics across the nation and the modeling of the costs and benefits of new clinic based services. This work examined the parameters and issues arising from the introduction of new services at these clinics. Boston IVF was one of the IVF sites in the study. The Study of Frozen Embryo Donation and Adoption Services is substantively the most relevant prior work done by the evaluation team.

h. Subject Characteristics

The research component of this Demonstration project is aimed at

1) Evaluating the impact of Boston IVF's new services designed to increase the donation of frozen embryos for use and to increase the number of recipients seeking to use this genetic material for family building. This element will use de-identified survey data.

2) Potential Donor families will be surveyed. Boson IVF, like all IVF clinics, stores the frozen embryos of their patients. Many of the families with embryos in storage intend to use them for family

building. However a number (estimated at 1200 families at Boston IVF) are “inactive”, that is, they no longer contemplate using their stored embryos. In general these are families who have ceased trying to build a family for one a several reasons and have not been in contact with Boston IVF for more than 12 months. These will be the study subjects. All such potential donors will be surveyed.

3) Potential Recipients of frozen embryos are the second study population. A number of Boston IVF patients enter the Clinics’ “Third Party Reproduction Program”. These families have stopped trying to have a children using the female’s eggs and are exploring alternative methods including egg donation and, under this Demonstration, the use of frozen donated embryos. Approximately 200 families are in this program at any time.

4) While not a primary study population the staff of the Boston IVF whose work is altered by this Demonstration will be interviewed on a limited basis as part of the effort to identify implementation issues important to the replication of the Demonstration.

i) Vulnerable Populations

All study subjects will be patients at the Boston IVF clinic and will be protected by Boston IVFs patient confidentiality rules. No identification of patients will be shared with the research team.

j. Recruitment

Details of the research protocols are presented below. In general all recruitment will be thru the Boston IVF and will be connected to the enhanced services authorized under this Demonstration. All interactions with patients will be via Boston IVF personnel and contacts will be made using present Boston IFV procedures in use as part of the expanded services being offered. Recipients will be surveyed as part of the intake process and information sharing done by the Third Party Reproduction Unit. As mentioned below all patients will be informed of their right to refuse to answer any research surveys.

Potential donors are routinely contacted each year by Boston IVF regarding the disposition of their frozen embryos. An enhanced range of alternatives will be offered to them under this Demonstration. Surveys will be conducted from Boston IVF lists to track attitudes and knowledge.

K/l. Study Design and Procedures

This is a Demonstration project and Boston IVF is the prime contractor. Its explicit goal is to test the practicality and impact of the expansion of IVF services to include offering IVF patients the opportunity to donate frozen embryos to other families for use in family building. It also includes efforts to offer this option to patients looking for a way of having a child using other people’s genetic material. Finally the Demonstration contains a research component whose goal is tracking the Demonstration and drawing conclusion from its operation. The Brandeis University research team is directly in charge of this component.

For completeness sake both the operational and research elements of the project will be explained.

<u>Project Aims</u>

This project is designed to test the efficacy of a new constellation of IVF center services in addressing both the shortage of donors and the problems of recruiting recipients. The key to the improvement in utilization of frozen embryos lies in increasing the involvement of the nation’s Assisted Reproductive Technology Centers. While these centers provide reproductive assistance to a limited percentage of the 6 million estimated infertile couples in the United States, they are the locus of all

activities directly capable of changing present patterns. **All** frozen embryos originate from their patients. They are, effectively, the only potential source of donated embryos. On the recipient side, it is their patients who are at the end stage of treatment options, who constitute the entirety of the candidates for the use of these embryos. Brandeis University and Boston IVF have formed a partnership to facilitate the availability and utilization of embryos in storage for procreative needs. The treatment or clinical service is called the Frozen Embryo Donation Service (FREDS).

<u>Model of the new procedures and services to optimize FREDS</u>

Embryo donation is not the first line of treatment raised when a clinical condition has the indication for the use of donated oocytes. The main focus of treatment is the use of donated oocytes, (egg donors) or adoption of a child or, finally, closure on family building. These treatments have clear methodologies, are universally accepted nation-wide and have developed organizations and protocols to which patients may turn.

In the current model, the embryo donation system is very underdeveloped. The patient who wishes embryo donation will need to research the inventory of a small number of agencies and ART centers that report their available frozen embryos for donation. A myriad of steps are needed such as registration, collection of medical data, history and various test results are needed before the patient knows whether they want any of the available embryos. The availability of actual supply of donated embryos is low and so access to them is very limited. The result is that the present system is, effectively, unavailable to many.

The Innovations of the new Model: Operational Elements of the Project Design

The availability of this grant will allow Boston IVF, in partnership with the researchers at Brandeis University, to develop a number of in-service trainings to inform and capacitate staff regarding the new services and tasks associated with donated embryos. These efforts will include:

1) Conducting motivational and educational sessions at Grand Rounds attended by approximately 25 providers of ART services. Educational efforts aimed a clinicians within the Center are critical if patients are to be communicated with effectively about embryo donation and use. The outcome of these sessions is to establish Frozen Embryo Donation as a realistic, seamless, cost effective and ultimately successful treatment indicated for patients regardless of marital status, ethnicity, sexual orientation, lifestyle in whom oocyte donation is indicated.

2) Conducting in-services and ISO 2009 organizational protocols for nursing staff in our 3rd party reproduction unit. These are the staff who will interact most directly with patients

3) Conducting mental health - social worker in-services on inclusion of embryo donation treatment in their counseling of patients undergoing treatment with donated gametes. Both potential donors and recipients of frozen donated embryos face unfamiliar and sensitive decisions. The mental health- social work staff would will provide the counseling that is necessary to help patients make these choices.

Boston IVF already has a well developed system for patient education and counseling and a strong system to staff training to support that system. These are additional elements to an on-going system.

<u>Donor Interventions</u>

The planned intervention will be aimed at non-current (inactive) patients of the Clinic who have embryos in frozen storage, as this is the pool from which donation can occur. Patients who have been in treatment within the last year will be excluded as this group is most likely to still intend to use their embryos and least likely to be ready to make embryo disposition decision.

From the eligible patients, a random sample for the intervention group will be selected. We estimate that about 1200 patients have embryos in storage at Boston IVF for more than one year. Six hundred will be randomly selected into the control group. The intervention group of 600 will be offered more proactive and costly services. These will be detailed below.

Baseline Practices

Upon storage of embryos all families are now asked to sign an embryo disposition form, which states the options open to them. The present form does not contain an explicit option regarding the donation of embryos for use by other families. This form will be revised during this Demonstration to include that option. This change is not expected to influence the Demonstration outcomes because patients receiving the new form will not fit patient selection criteria during the data collection phase of this work. That is, this will affect only new patients who could not have embryos in long term storage. This change is being instituted as a best practice/ service to all patients.

Presently the Clinic contacts inactive patients with embryos in storage once a year around the process of billing for embryo storage. The yearly storage fee is $600 and bills are sent out on a rolling basis on the anniversary of the storage placement. Prior to the bill, a letter is sent saying that the anniversary is coming up and reminding them that a decision regarding embryo disposition will have to be made- even if it's only continued storage. No details or recommendations are included. No specific contact information is included and no proactive contacts with families are undertaken. group. For the control group no change in current practice will occur.

Description of Intervention

Families in the intervention group will receive a different letter at billing time from that sent to the control group. This letter will urge them to consider the embryo disposition options. It will include a focus on the altruistic character of donation. The letter will include several options for going forward including an invitation to speak with the doctor or nurse in their care team. In it a named nurse, who is in their care team, will be identified, along with contract information. Patients will be told of the availability of group education sessions and of one on one counseling/information sessions. A free visit with the Center's social work staff will also be offered**.** It will also be mentioned that, once donated, the patients will no longer be responsible for the embryo storage fee. All responses will be logged for later tabulation and analysis. (See evaluation section below).

In addition to the letter the intervention group will receive a telephone follow-up. It will ask if the letter was received and to see if the family members have any interest in additional information. As before, all responses will be logged for later tabulation and analysis. We will manage and track the outreach letters, respond to any call backs etc. Staff will receive training on the technical questions regarding embryo donation and the potential use of donated embryos. If needed, additional training regarding patient interaction will be provided, although these staff are experienced in these matters as part of their present patient interactions through the Third Party Reproductive Unit. The research team will provide log sheets to gather relevant data on family responses and methods for coding family questions and concerns. These data will be de-identified prior to sharing them with the research team.

The Clinic will develop protocols for both the group education meetings and the individual counseling sessions. Separate protocols will be developed for telephone conversation and for face to face sessions. The educational meeting will be designed to provide information on alternatives open to patients, the need for donated embryos and the process for making the donation. A primary element in this regard will be making the process simple and quick for the patient. Presenters will provide ample time for questions. The option of continuing questions and/or concrete discussions on a private session will be emphasized.

Recipient Focused Services

The goal is to develop, implement and evaluate clinic services designed to offer these embryos of patients using the clinic for reproductive assistance. This will involve development of educational materials, provision of assistance and counseling, provision of technical medical and test services and tracking outcomes of these efforts.

During the Demonstration, present clinical embryo donation practices will be continued but on a larger scale. Under present practice, a candidate recipient will schedule an initial consultation with one of the two BIVF FREDS physicians, Drs. Oskowitz and Zimon. The consultation includes a complete review of the candidate's medical and reproductive history. Under the Demonstration an assessment of the indication for FREDS will be made. And, an overview of the FREDS program will be provided, including all procedures, testing, protocols, and requirements for the patient to complete a FREDS recipient cycle. This will include a discussion of options for donated embryo sources including third party agencies or known donations. In addition, alternative options for building a family will be discussed as applicable including third party reproduction through donated eggs, donated sperm as well as adoption. The FREDS program is aimed at expanding the services that can be offered to recipients, not to replace existing alternatives.

In the next stage, comprehensive testing and consultation to ensure medical suitability for embryo transfer and pregnancy are completed. Complete medical, gynecological, obstetrical and genetic histories are reviewed. These are standard elements in the medical protocols of Boston IVF. An additional review of health care maintenance testing and vaccinations in accordance for national guidelines is performed and deficiencies are addressed. Standard pre-conceptional testing and screening is performed including transmissible disease screening, blood typing and blood antibody testing, and immunity screening. These practices will not change during the Demonstration.

At the present time, Boston IVF does not have a donor recipient matching program, nor does it have a pool of embryo donors or banked donor embryos. Therefore, currently, candidate FREDS recipients must match to embryo donors independently or via a third party agency. One of the goals of the Demonstration is to develop such a pool and a matching program or to explore links with existing programs. (See donor recruitment section.)

Typically, it is only after the embryos have been donated that the protected health information and data pertaining to the donors and the embryos is provided for review of professionals at Boston IVF. At this stage, the donated embryos are assessed for compatibility with Boston IVF's laboratory protocols, overall quality, potential for pregnancy, and risks with respect to genetic or medical concerns. Therefore Boston IVF already has the technical expertise and protocol to do this work. During the demonstration, the embryo assessment will precede the matching and donation event to better serve the needs of the recipients.

Once a candidate recipient has identified potential embryo donors then the Boston IVF program ensures provides patients with a checklist of requirements and the support and referrals to complete all requirements. The initial steps for recipients include consultation with a physician at Boston, completion of all indicated testing, completion of referral consultations and counseling, consult with a Boston IVF financial coordinator and complete a financial agreement with or without insurance coverage. Next, patients identify source of donated embryos with or without use of a third-party agency or IVF center, compete a donor to recipient matching event as applicable, obtain legal counsel, draft and complete a legally sound embryo donation agreement, ascertain embryo assessment and compatibility with Boston IVF professionals, and arrange for embryo transport to Boston IVF. Finally, patients consult with their physician to finalize a treatment protocol and plan for transfer of donated embryos, provide written consent to proceed, and proceed with treatment cycle and embryo transfer.

Changes with the Demonstration

The Demonstration services outlined below will be offered as an option to all patients being served by the Center's Third Party Reproduction unit. That is, it will be offered to all Boston IVF patients considering terminating traditional fertility treatment, considering third party reproduction via donated eggs or other alternatives. Therefore there will be, unlike under the donor services, no control group. However, please note that the evaluation of these services will include a survey of eligible patients both before and after the new recipient services are instituted. This will allow a pre- post evaluation model.

The Boston IVF Center will develop professional recommendations and designate a "recipient" team. It has chosen Oskowitz and Zimon (Project PI) as the two clinicians. They will develop their plans based on literature, practice elsewhere, and professional norms. They will share the protocol with other clinicians and offer their colleagues the option of referring patients interested in this option to them, or offering them direct support. Efforts will be increased to assist clinicians to recruit patients to consider FREDS. This will include educating BIVF MDs to include FREDS as an option to all potential candidates, developing informational brochures and materials, expanding existing information available on the Boston IVF web page hosting informational seminars to further increase awareness of the program, and considering further outreach beyond BIVF by presenting educational seminars or lectures with advocacy or support organizations such as Resolve.

Through the Demonstration, the Third Party Reproduction program will be expanded to incorporate FREDS. Presently, this unit deals with patients who are no longer attempting to start a pregnancy solely with their own genetic material and services approximately 200 patients each year. About 150 of these patients receive a donor egg for implantation. Fifty do not end up going in that direction and either end their search for reproductive help or seek an adaption. An additional number, presently untracked, express an interest in third part reproduction but do not pursue the alternative formally. All patients in the Third Party Reproduction unit will receive new services under this project at no additional cost.

During the Demonstration, all written materials offered to these patients in this unit will be amended to include information on the possible alternative of using frozen donated embryos. The educational and orientation sessions will also be amended for this purpose. Additional group informational session will be offered to those interested in information about donated frozen embryos. These sessions will include clinical information, outcome rates, timing issues, costs and legal issues. For interested patients, individual counseling sessions will be offered to patients. These will be designed to provide detailed information about process, costs, medical procedural changes tailored to the particular clinical circumstances of individual patients, offer counseling on family and person issues dealing with relationships with donor families, confidentiality issues, dealing with issues of non-genetically related offspring. These may draw on experience of adoption agencies.

Another major effort of the Demonstration will be to develop streamlined and faultless clinical protocols for candidate FREDs recipients. ISO (International Organization for Standardization)-approved work instructions will be developed for all aspects management for physicians and nursing staff. Further, education and training will enable all will be expanded to BIVF physician care teams to shoulder FREDS recipient treatment management. This will be of great benefit for patients who will no longer need to seek consultation with a new doctor for a service provided at Boston IVF.

Under the demonstration, the scientific laboratory services will adapt a protocol for FREDS. An embryo assessment tool or protocol will be developed. Through this instrument, a designated embryologist will review available records and available on the donated embryos including quantity, quality, ART procedures performed, protocols for culture and freezing, and outcomes data for embryos from the specific donors. A specifically named Boston IVF embryologist will oversee the execution of this protocol and ensure training of all staff embryologists for function in this role.

The embryologist will contact staff at the source embryology laboratory of the embryos to verify embryo quality assessment and compatibility with Boston IVF's laboratory procedures. The embryologist will then provide an assessment of the suitability of the embryos for donation and an estimation of their potential to survive thaw and result in pregnancy.

The Boston IVF Center will assume, by the Demonstration funding, responsibility for all required donor family testing. Neither Recipients nor donors will, during the Demonstration, be asked to bear those costs. Laboratory costs associated with testing donor embryos will be a recipient responsibility. A Boston IVF financial coordinator will consult with participates to inform them of required fees and health insurance coverage if any.

During the demonstration, legal and social counseling services will be expanded. Consultation with a mental health professional regarding the use of donated embryos will be required for all FREDS recipients. Three licensed social workers are on staff at Boston IVF and will provide these services for patients without charge. At the present time, legal counsel and arriving at the legal donation agreement is outsourced independently, either via a third-party donation agency or an independent attorney. Under the demonstration, a protocol will be developed, similar to what is in place for third-party reproduction services of donated egg and gestational carrier, to facilitate access to legal counsel and drafting of a legal donation agreement.

<u>Operational and Organizational Elements of the Demonstration</u>

Adding an additional component to the services offered patients at the Boston IVF center will require a number of administrative changes both to support the new services and to allow the evaluation of the demonstration over-all. The needed demonstration services (see Figure 2) are extensions of the on-going systems at the clinic but go beyond them in 3 general areas: (1) Staff training to insure knowledge about the frozen embryo program and in insure consistency in communications with patients; (2) The development of documents, protocols and procedures needed to manage and offer the new services; (3) Information system elements to track the Demonstration.

Staff Training

While the counseling and social service staff of the Clinic are well trained in educational and counseling services, they will need additional training of the key elements of the Demonstration effort. This will require factual training on the issues arising from the use of frozen embryos, including such things as timing, costs, legal issues etc. It may also require additional training on the psycho-social issues that arise about this technology. A member of the counseling staff will spend the early months of the Demonstration educating herself about these issues and identifying additional local resources if they are needed. The Brandeis University research staff will assist in this activity. Other staff members will also need training. Changes in laboratory practices may require training for the nursing and laboratory staff. Most clinicians in routine contact with patients will need to be informed about the new program, key communication issues and the internal division of responsibilities so they are refer patients to the proper people. In the same way receptionist and telephone staff will need to be informed about new protocols and the transfer of patients' calls to the Demonstration staff.

The outreach efforts to donors will be a new responsibility. A staff member with counseling experience will be selected for this task but additional training may be called for. As part of this person's duties will include data recording, specifically patient responses, the person involved will have to be trained for this task. The Brandeis University staff will conduct this training.

Development of Documentation and information systems:

A number of new documents and procedures will have to be developed for the Demonstration. Most will involve modification of existing materials. In addition to training and presentation materials, discussed above, the following new materials will be developed: (1) Existing permission and consent forms for donors will need to be updated to include the donation of frozen embryos, the legal ramifications of donation and the financial ramifications- once a donation decision is made the Demonstration, not the family, will be responsible for storage costs; (2) Existing permission and consent forms for potential recipients will need to be modified; (3) Laboratory protocols for testing the donated frozen embryos will need to be developed; (4) Protocols relating to FDA requirements for donation safely will need to be developed.

Information System Modifications

Both the expanded services and the evaluation process will require new information being gathered and maintained. Some of these new demands are: (1) The shift of financial responsibility from patient to the clinic for embryo storage must be done in a timely manner and patients must be kept informed; (2) As the Demonstration includes a cost/benefit analysis the internal costs of the program will have to be tracked by the Clinic systems; (3) Data on patients in the intervention group will be separated into a data system insulated from the Clinic's general data system to protect confidentiality and to allow the tracking of patient responses to the intervention. This data set will be used for tracking the intervention (letters and telephone follow-ups), and patient responses, ranging from calls to the Clinic to embryo disposition decisions. These data will need personal identifiers for Clinic activities and will need to be de-personalized for research purposes; (4) Logs and records will be developed to allow the tracking of communications to staff, educations sessions etc. This is to help facilitate the process evaluation of the demonstration.

Evaluation

Evaluation Plan for Donor Services

The evaluation seeks to answer three key questions around eligible donors: (1) Baseline attitudes and needs of eligible donors and their implications for program design; (2) Program impact on attitudes, experiences and behaviors of the intervention group compared to the control group; (3) Program costs and cost-effectiveness.

Baseline Attitudes and Information Needs

We will conduct a baseline survey of a 50% sample of the 1200 eligible donors. The instrument to be used will be developed during the initial stages of the project. The target population is former patients who have frozen embryos in storage and who have not been in active treatment for the past year. The proposed consent procedure for this and all surveys will be a cover letter explaining that all survey responses will be confidential and used only for statistical analysis. Identified responses will not be shared with any clinical personnel or outside personnel. Furthermore, response to all surveys overall and to individual items is strictly voluntary. Neither the decision to respond nor any information provided will affect any future care or payments at Boston IVF.

Topics will include basic demographics (e.g., age, education), past experiences with fertility treatment and outcomes, knowledge about embryo donation, gamete material in the embryos (i.e., source of eggs and sperm), and attitudes towards embryo donation. Many attitudinal questions will be asked the in theform of reaction to statement, such as "I am very interested in donating my frozen embryos for use by another patient for the purpose of having a child." Responses will be requested using a 5-point Likert scale from "strongly disagree" to "strongly agree". This information will be used to identify the types of patients who might be most inclined towards donation so interventions could be targeted and framed most appropriately. The survey will show the knowledge that patients

already have and the gaps that exist so that educational brochures and preparation for telephone calls and face-to-face meetings will be most appropriate. All surveys will be deliberately short (no more than both sides of one page) to minimize time demands on participants and to maximize response rates.

We have used a sample rate of 50% to allow enough precision to be able to measure changes at follow up while minimizing the risk that the baseline survey itself sensitizes the eligible population about embryo donation becomes an intervention that could not be evaluated. At follow up, we will be able to assess changes in attitudes due to the survey and each of the interventions compared to the control group.

Program Impact

We are interested in knowing how eligible patients felt about being offered intervention services, and what, if any, impact these services had on their subsequent behaviors. Previous efforts around awareness and donation services were conducted in an uncontrolled environment, which makes separating effects of any specific program from changes other factors (e.g. the health care system, economy, technology, insurance coverage, and national attitudes) problematic. Here we propose a randomized controlled design to address this challenge. Short term impacts will be assessed through a 90-day follow up survey. With the same protections as described above, patients will be asked what intervention services they recall receiving and their reactions around each service – whether they considered the services respectful and helpful. We will be able to determine how responses differ by patient and intervention characteristics – such as whether patients with embryos in storage for a longer period were more favorably inclined, and whether the phone calls in the more proactive intervention group were considered beneficial or intrusive. Sample questions will be: "I thought the communications from Boston IVF (letters and/or phone calls) about embryo disposition were helpful," and, as on the baseline survey "I am very interested in donating my frozen embryos for use by another patient for the purpose of having a child." Again, responses will be on a 5-point Likert scale. As the survey comes from a known, respected organization with which most patients have had an intensive relationship, we anticipate a response rate of 75%, so that we expect 300 respondents per arm. One of our key items will be a favorable attitude towards embryo donation (4 or 5 on the 5-point Likert scale). Past research has previously found low interest. We anticipate a 15% favorable rate in the low intensity intervention group and a 25% favorable rate in the proactive group. Sample size calculations show that these assumptions require a sample size of 289 (which we rounded to 300) subjects per group under standard conditions of a significance of 5% and a power 80% (See Fleiss JL, Statistical Methods for Rates and Proportions, Wiley, 1973). Thus, our sample will be sufficient to generate significant differences between the two intensities of intervention using these dichotomous outcomes, and even more sensitive using multivariable approaches described below.

A secondary analysis will compare respondents within each arm who received the baseline survey with those who did not. This will allow us to determine whether even a modest item to raise awareness by asking questions changes attitudes – an important finding for future awareness efforts. The advantage of this design is that we are measuring awareness in the population for whom it is most relevant – eligible patients who have embryos in storage and are potentially in the position to donate them. These analyses will be conducted using multivariable regression as a more sensitive approach, where the dependent variable is the average of the Likert scale on several related items, which we will group and weight using Principal Components Analysis.

The major measure of impact will be final attitudes one year after intervention. That will be assessed through the follow up survey of eligible patients at one year, including the control group, thus allowing controls for unrelated trends. As our total sample of 600 is divided between two arms, we will have 300 subjects per arm. Again assuming a 50% response rate, we anticipate 150 respondents per arm. As more time will have elapsed and the intervention groups will have had more opportunity to receive services, we anticipate further improvements the favorable ratings in those two groups, with

anticipated favorable rates of 5% in the control group, 20% in the low intensity intervention group, and 36% the high intensity group. Using the same statistical assumptions, the required sample sizes to compare the two groups are 121 subjects per group. Thus, our expected sample size of 150 per arm is sufficient to detect the hypothesized differences. We will also do further multivariable regression analyses with the actual Likert scale as a continuous variable which will be more precise, allow us to distinguish effects by baseline characteristics, responses to the baseline survey and other covariates, and produce significant effects even if the response rate or differences were lower than projected.

A secondary set of outcomes through program data will assess patient behavior and compare among the randomized arms. Boston IVF tracks phone calls with clients and face-to-face encounters. Thus, we will be able to determine the proportion of patients in each arm that has any documented discussion about embryo disposition with Boston IVF staff, the proportion who have an in-person encounter, and the proportion who actually proceed with donation. While the latter numbers may be small within the one-year window, they become the most important, objective evidence of these interventions.

Cost and cost-effectiveness analysis

The costs of intervention services will be assessed by determining the time of staff spent developing and delivering intervention services, plus other direct expenses, such as printing, mailing, legal and laboratory fees. These will assessed through documents from the Boston IVF financial office. We will not count the development nor evaluation elements of the budget. The key analytical steps will be estimating two types of incremental costs. We will assess how the cost per patient randomized compared between the control and intervention groups. The cost effectiveness will be based on an incremental cost effectiveness ratio, defined as: Incremental cost of an intervention divided by incremental effectiveness the intervention.

The effectiveness and cost-effectiveness results will be used by Boston IVF and disseminated according to the dissemination plan for use by other clinics around the country.

<u>Evaluation Plan for Recipient Services</u>

The recipient target population for these services shall be all patients of Boston IVF seeking service in the third party reproduction unit. There are approximately 200 patients receiving services in this way as of June 2011. Approximately 150 enter this service each year. Patients in this unit have given up of their efforts to become pregnant using their own genetic material and are now considering alternatives. They are, therefore, the population from which demand for frozen donated embryos must come. Their attitudes and preferences are therefore, the most relevant grounds for estimating demand.

The central goals of the evaluation of the recipient based services provided under this grant are: (1) To estimate the demand of donated frozen embryos; (2) To determine the information and beliefs regarding the use and availability of donated frozen embryos; (3) To determine the factors that play a role in recipient decision making regarding alternative third party approaches to IVF; (4) To determine recipients' responses to the Clinics new frozen donated embryo services and education efforts.

The primary evaluation tools to be used are patient surveys. A survey of all patients using the Clinic's third party reproductive services will be conducted in month 3 of the project. The same survey questionnaire will be offered to newly joining patients during the duration of the data gathering period. We therefore estimate a total survey sample of about 300. These surveys will be conducted before patients are exposed to any new services.

The survey will use Likert scales to probe: (1) Patients' knowledge regarding donated frozen embryos. What information patients would like to have regarding this alternative; (2) Patients' attitudes regarding this option; (3) Factors that make frozen embryos an attractive or less attractive alternative compared to egg donation or adoption.

Among patients who do not available themselves of the educational and counseling services offered under this Demonstration, the survey will be repeated during a regular visit to the clinic approximately six months after the patient's first exposure to the newly developed standard orientation meeting. (As part of this project, that orientation will be modified to touch upon the frozen donated embryo option.)

This second survey will allow us to track attitudes over time and provide information about the retention of information provided during initial orientation. Long-term impacts of the new orientation are more important for the Demonstration than the immediate impact post educational session. Therefore the follow-up survey will not be done immediately after exposure to the new educational presentation but several months later. These surveys will provide baseline data on the knowledge and concerns of the pool of patients most likely to consider frozen donated embryos as a solution to their reproductive problems. And, within the limits a single site-based survey, add to our knowledge about demand for these services among a pre-intervention population and after a minimal intervention.

Most patients will not want any additional services beyond that contained in the new orientation. For those who available themselves of the more intensive recipient services there is an additional evaluation element. As discussed, these services will include group educational sessions and individual counseling sessions. The group sessions will be attended by a member of the research staff and the questions asked by recipients will be noted and then coded. No patients will be identified. This work will employ non-participant observational techniques.

The general issues arising in the private counseling sessions will be accessed via face to face interviews with counselors. Here again these interviews will be general in nature and not related to any individual patients. A semi- structured interview guide will be used for the counselor interviews using methodologies recommended by Dr. Yin and Drs. John and Lyn Lofland. (Robert Yin, Case Study Research, Sage Publications, 2003 and Lofland and Loflan, Analyzing Social Settings, Wadsworth Publishing, 1995)

In this approach, broad questions are prepared with a number of additional "probe questions' held in reserve. Probe questions reflect the theories underlying the analysis. They are not actually asked of the respondents if the respondent spontaneously provides the information sought. However if that information is not provided then the probe questions are asked. This allows the research to benefit from both a semi-structured interview model, which allows maximum input from knowledgeable respondents, and, at the same time, it insures the consistency and completeness of information gathering characteristic of a structured interview questionnaire.

Documentary data will also be gathered to track: (1) The number of patients seeking to use frozen donated embryos; (2) the number of patients who contacted the Clinic for additional information about the use of frozen embryos; (3) the number of patients seeking one on one information and counseling, and (4) the number of patients attending educational service.

These data will be used, along with the survey data, to estimate the impacts of the intervention. The survey data will provide attitudinal measures, the documentary data will track patient behavior from the most complete (and the deseratium) of selecting to use donated frozen embryos, to the least change, attending informational sessions.

M. Risks and Benefits to Subjects

Subject will be at no enhanced risks. The operational aspects of the projects involve a small extension of Boston IVF services. Both donors and recipients may benefit if they chose to available themselves of the additional opportunities offers. Recipients may find family building more available and less expensive.

Donors will have an opportunity to use stored embryo to help other families and, secondarily, the opportunity to avoid the cost of embryo storage.

The data collection efforts will invite them to give their views on a number of matters generally of interest to patients in IVF treatment. The confidentiality protections and their absolute right to decline participation will ensure they are at no risk.

n. Managing risk

The de-identification of all data, confidentiality assurance and informed consent will all be used to insure the protection of subjects.

0. Cost and compensation

Strictly speaking participants will be subject to neither by the research protocols. The enhanced services of Boston IVF under this Demonstration may save some patients the costs of embryo storage.

P. Informed consent

Informed consent is a central aspect of Boston IVF practices as it is for all routine medical treatments. Additional inform consent protocols are associated with each data collection element. Data will be collected from Boston IVDF patients by mailed surveys in the case of potential donors and in-person written surveys or mailed surveys for recipients. In-person written surveys will be offered to recipients during intake and educational events in the Third Party Reproduction Unit by Boston IVF staff. In all instance the informed consent and the right to decline material will be part of the documentation offered to subjects. The preliminary informed consent document is appended.

q. Data storage

Survey data will only arrive at Brandeis without identifiers and will be stored in password protected computer files. It will be saved only until the project's end and will be accessible only to Brandeis researchers conducting the project.

Confidentiality and Consent Form

Preliminary Consent Form;

The goals of this study are to identify what patients at ART clinics know about the potential uses of frozen embryos and to examine attitudes toward the donation and use of such embryos. A sample of Boston IVF patients have been randomly selected to receive this questionnaire. You are being asked to assist in this research because of your experiences as a patient of the Boston IVF clinic. All information collected in this study is confidential. The data you provide will be grouped with data others provide for the purpose of reporting and presentation. No information you provide will be associated with your name.

You are not, of course, obligated to participate in this research. Your participation or your decision not to participate will have no effect of any sort on your relationship with Boston IVF. If you understand these conditions and are willing to be interviewed please sign below.

Survey Instruments

These are in development during the first months of the project.

STUDY PROTOCOL

Part 2

Modification submitted June 2013

Memorandum

To: Brandeis University Committee for the Protection of Human Subjects (IRB)
From: Donald Shepard

Subject: Requested modification for RB Protocol #12032 Provision of Frozen Embryo Donation Services (FREDS) in a Large IVF Facility

cc: Jeffrey Prottas, Alison Zimon, Denny Sakkas, Selwyn Oskowitz, Clare Hurley

Date: June 4, 2013

My co-investigators and I are writing to notify the Brandeis IRB about the suspension of some study elements, to request modifications and renewal, and to add personnel for "IRB Protocol #12032 Provision of Frozen Embryo Donation Services (FREDS) in a Large IVF". The requested modification in procedures consists of some alterations in the sample selection/ intervention model, a change in the intervention letter that reflects that change, and a data set that allows us to correct for possible response bias if necessary.

1) Suspensions: The original intervention model planned three study arms: a control group, a low intensity intervention group and a higher intensity intervention group. We have suspended the low-intensity intervention arm, so our study now has two arms. We have also limited our present analysis to patients in both arms who responded to an initial baseline survey, as these data were necessary for the evaluation. These steps were taken because the baseline survey indicated a lower degree of interest in considering embryo donation than we had anticipated. This result indicated that the low intensity intervention was highly unlikely to have any measurable impact on attitudes. This has reduced the overall sample size but increased the contrast between arms being compared. As we are merely suspending items previously approved by the IRB, these items are notifications, rather than requests for approval.

2) Proposed changes in use of intervention letter: As a result of the suspensions and to simplify the operational demands on Boston IVF, we plan to alter the way the intervention letter will be used. Each month, Boston IVF identifies patients who will reaching the one-year anniversary of their storing embryos three months in the future. It sends them a notice that they will receiving a bill for annual storage in three months' time if they continue to have embryos in storage at that time. For patients who still have embryos in storage at the one year anniversary, it sends the annual bill.

   We propose to use this study to randomize patients equally between intervention and control groups. The control group would continue treatment as usual. The intervention group will be sent the letter offering them a free counseling session. The letter is enclosed and, as before, invites the recipient to contact a professional at Boston IVF for more information. In addition, when patients in the intervention arm receive their yearly storage bill the intervention letter will be included again for patients in the intervention group who have not

responded based on the three-month notice. This is a routine communication from Boston IVF to patients and is, therefore, operationally simple to do and likely to be read.

The letter is very similar to those sent previously, but there have been wording changes to reflect that way it is being send out and the lessons regarding terminology learned during the pre-test and fielding of the background survey.

3) Characteristics of respondents and non-respondents to survey of patients. As the response to date to the survey of Boston IVF patients is only about 25%, it will be important to understand how respondents compare to non-respondents and to be able to adjust for possible biases. For that reason, we request permission for transfer from Boston IVF to Brandeis of a limited data set on Boston IVF patients who received a survey about attitudes towards embryo donation. Brandeis will agree to maintain the privacy and confidentiality of this data set, avoid trying to identify any subject, and to use it only for mutually agreed research projects. The main variables in the limited data are the following:
    a. Internal study sequence number
    b. Year of birth
    c. Source of female gametes (patient or other)
    d. Most recent embryo freezing (month and year)
    e. Has IVF treatment resulted in a pregnancy (yes/no/unknown)
    f. Has IVF treatment resulted in a pregnancy and live birth (yes/pregnancy but no birth/no/unknown)

[Boston IVF letterhead]

Dear Patient,

We are contacting you as part of an initiative to support patients who have frozen embryos stored with Boston IVF.

You may be planning to use these embryos in the future, in which case you have likely decided to continue ongoing storage. If you are not planning to use your embryos in the future or have not yet made a decision, you may know that your options include ongoing storage, discard, donation to other individuals or families, or research donation. We recognize that these decisions are complex and information regarding your options may be difficult to obtain.

This service was initiated with the support of a grant from the U.S. Department of Health and Human Services (HHS). **We invite you to meet with the Director of Boston IVF's Counseling Services to explore and understand your options with respect to your frozen embryos. This consultation is free of charge.**

The consultation is conducted by Jeanie Ungerleider, LICSW, BCD, who is Boston IVF's Director of Counseling Services and has over twenty-five years of experience working with fertility patients at Boston IVF. The objective is to provide you with information and resources regarding embryo disposition as well as the support to deal with the difficult decisions regarding your embryos. Other Boston IVF patients have found these consultations extremely rewarding. Though this service has support from the Department of Health and Human Services, **it is not a government project** and the government will not receive any personal information about participants. There is no obligation to participate and your participation has no bearing on the status of your embryos or associated notifications or fees. This initiative will cover embryo storage fees going forward should you choose to donate your embryos to other individuals or families.

If you are interested in learning more about this initiative, please contact our **Program Coordinator at Boston IVF, Kristin Rooney, at 781-434-6470 between 7am and 3pm weekdays**. As always, your Boston IVF physician and team are available for support and information if you wish to further discuss your frozen embryo options.

Sincerely,

Selwyn P. Oskowitz, MD
Alison E. Zimon MD
Boston IVF Physicians
