# Supplementary material for: Embryo donation: Survey of in-vitro fertilization (IVF) patients and randomized trial of complimentary counseling
Source: PLoS One. 2019 Aug 15;14(8):e0221149. doi: 10.1371/journal.pone.0221149 (PMC6695140; doi:10.1371/journal.pone.0221149)
Supplement: S10 File — This letter to Boston IVF patients with frozen embryos in storage, instituted after the trial, informs them about the availability of a complimentary counseling session. (DOCX) [file pone.0221149.s010.docx]

Dear Boston IVF patient,

We have enclosed this letter as a reminder that you currently have embryos frozen and stored at Boston IVF.  Your yearly storage fee for your embryos will be submitted on [date].

It is our hope that you will choose to use these frozen embryos in the near future. We recognize, however, that many of our patients would like to continue to maintain the embryos in storage for future attempts at pregnancy, while others have completed their families and would like to make arrangements for disposition of their embryos.  We provide counseling services for decision making and offer a complimentary initial consult with an experienced mental health professional.  If interested, please call [name and phone of counselor].

We ask at this time that you review your plans for family building and make a decision regarding embryo disposition. We would like you to consider the following options:

1.            Continue embryo storage at Boston IVF and accrue monthly storage fees.

                                 Important notice regarding storage fees:

Boston IVF will be [fees].

2.            If you decide you are ready to arrange to discard your embryos you will need to complete, notarize and return an Embryo discard consent.

a.            All forms can be found at our website bostonivf.com.

b.            Click the option "Patient Portal" located at the bottom of the page.

c.             Choose the option "Patient Forms"

d.            Click on the Lab consent tab

e.            Download “Discard Frozen Embryos Consent”

f.             Complete and return the consent to:

Boston IVF

Embryology Lab

[Mailing address]

3.   If you decide you would like to donate your embryos to scientific research or relocate the embryos in storage please call [contact person].  Not all patient’s embryos qualify for donation.

4. Go Green!  Save time, money and postage for future storage billings - complete the recurring payment form attached to have monthly storage bills [processed automatically].

We will need a completed recurring authorization form and your credit card information to enroll you in the automatic payment program.  The completed form must be received prior to the 1st of the month your billing begins.  Please mail the form to:

Boston IVF

[mailing address]

We realize that the decision regarding embryo disposition can be a difficult one; we have allowed 90 days for you to make a decision before incurring any costs.  We recommend that you contact your Boston IVF physician to discuss your options.

Storage fees will continue until discarding or donating paperwork is received.

Thank you in advance for choosing to work with the physicians and staff at Boston IVF, the Best Fertility Experience.

Recurring Payment Credit Card Authorization

The undersigned authorizes Boston IVF to charge my credit card for recurring the Payment Plan Payments I have agreed to.  The following conditions apply to the recurring payments program:

I, _________________________, have entered into a payment plan arrangement with Boston IVF for a total of $XX, payable in recurring monthly payments of $XX.

Please fill out this form COMPLETELY

Patient Name: __________________________________________Date of Birth: ____________________

Billing Address: ____________________________________________ Phone #:____________________

City, State, Zip_________________________________________________________________________

Signature:  __________________________________________________Date:_____________________

Payment Authorization
