# Supplementary material for: Embryo donation: Survey of in-vitro fertilization (IVF) patients and randomized trial of complimentary counseling
Source: PLoS One. 2019 Aug 15;14(8):e0221149. doi: 10.1371/journal.pone.0221149 (PMC6695140; doi:10.1371/journal.pone.0221149)
Supplement: S4 File — This questionnaire was mailed to sampled patients behind the cover letter in S3. (DOCX) [file pone.0221149.s004.docx]

**Confidential Survey of Boston IVF Patients**

***Please use the following 5 point scale and circle the response that best applies.***

| **A.** | **In terms of how you feel right now, which options**  **would you choose for your frozen embryos?** | *Strongly disagree* | | *Don’t know*  *(Neutral)* | | | *Strongly agree* | |
| --- | --- | --- | --- | --- | --- | --- | --- | --- |
| **[1]** | Store for future attempts. | **1** | **2** | | **3** | **4** | | **5** |
| **[2]** | Thaw and dispose of embryos. | **1** | **2** | | **3** | **4** | | **5** |
| **[3]** | Donate to another couple trying to have a baby. | **1** | **2** | | **3** | **4** | | **5** |
| **[4]** | Keep the embryos frozen for a later decision. | **1** | **2** | | **3** | **4** | | **5** |
| **[5]** | Donate the embryos to research. | **1** | **2** | | **3** | **4** | | **5** |

| **B.** | **I might be more willing to donate my stored embryos to another Individual or family if:** | *Strongly disagree* | | *Don’t know*  *(Neutral)* | | | *Strongly agree* | |
| --- | --- | --- | --- | --- | --- | --- | --- | --- |
| **[1]** | I was sure I did not want more children. | **1** | **2** | | **3** | **4** | | **5** |
| **[2]** | I know who would be using my embryos and could speak with them. | **1** | **2** | | **3** | **4** | | **5** |
| **[3]** | I had the opportunity to know basic characteristics (e.g., age, religion) about the recipient individual or family. | **1** | **2** | | **3** | **4** | | **5** |
| **[4]** | The recipients live in a different area or state. | **1** | **2** | | **3** | **4** | | **5** |
| **[5]** | I could be informed of the outcome of the embryo transfer, such as pregnancy and live birth. | **1** | **2** | | **3** | **4** | | **5** |
| **[6]** | I understood the legal implications. | **1** | **2** | | **3** | **4** | | **5** |
| **[7]** | I could speak to a counselor. | **1** | **2** | | **3** | **4** | | **5** |

| **C.** | **Thinking about embryo donation:** | | *Strongly disagree* | | | | | *Don’t know*  *(Neutral)* | | | | | *Strongly agree* | | | |
| --- | --- | --- | --- | --- | --- | --- | --- | --- | --- | --- | --- | --- | --- | --- | --- | --- |
| **[1]** | Embryo donation is a way to give an embryo  a chance at life. | | **1** | | **2** | | | | **3** | | | **4** | | | **5** | |
| **[2]** | Embryo donation is a way to help another couple. | | **1** | | **2** | | | | **3** | | | **4** | | | **5** | |
| **[3]** | Embryo donation has many similarities to the placement of  a child for adoption. | | **1** | | **2** | | | | **3** | | | **4** | | | **5** | |
|  |  | |  | |  | | | |  | | |  | | |  | |
|  |  | |  | |  | | | |  | | |  | | |  | |
|  |  | |  | |  | | | |  | | |  | | |  | |
| **C.** | **(Cont’d) Thinking about embryo donation:** | *Strongly disagree* | | | | *Don’t know*  *(Neutral)* | | | | | *Strongly agree* | | | | |  |
|  |  |  | |  | | |  | | |  | | | |  | |  |
| **[7]** | If I donated embryos, I would be uncomfortable with the idea that someone other than me would raise my genetic child. | **1** | | **2** | | | **3** | | | **4** | | | | **5** | |  |
| **[8]** | Donating a stored embryo to a family for family building is complex and difficult | **1** | | **2** | | | **3** | | | **4** | | | | **5** | |  |
| **[9]** | I am concerned that there might be expenses involved in being a donor | **1** | | **2** | | | **3** | | | **4** | | | | **5** | |  |
| **[10]** | I am concerned that I might have to undergo testing or medical procedures to be a donor. | **1** | | **2** | | | **3** | | | **4** | | | | **5** | |  |

**D.	Demographic information. *Please circle the response that best applies.***

| **[1]** | I am: | Female | Male |  |  |  |
| --- | --- | --- | --- | --- | --- | --- |
| **[2]** | I have: | No children | 1 child | 2 children | 3 or more children |  |
| **[3]** | I used donor gametes to create embryos during IVF: | Yes | No |  |  |  |
|  | If yes, I used: | Donor sperm | Donor eggs |  |  |  |
| **[4]** | My highest level of education is: | High school or n/a. | Some college or equivalent | College graduate | Graduate degree |  |
| **[5]** | My religious tradition is: | Protestant | Catholic | Jewish | Other  (*please specify*): | |
| **[6]** | I identify my ethnicity as: | White non-Hispanic | Hispanic | African-American | Asian | Other (*please specify*): |
| **[7]** | My experience with assisted reproductive technology (ART) has been: | Largely negative | Somewhat negative | Neither positive or negative | Somewhat positive | Largely positive |
| **[8]** | Overall my partner would agree with me on the responses on this survey. | Yes | Not sure | No |  |  |

**E. Additional Information**

The disposition of stored embryos is a complex decision. A survey such as this one inevitably misses much. If you have any other thoughts about embryo donation, please feel free to write them below.
